# Semantic-Attention Enhanced DSC-Transformer for Lymph Node Ultrasound Classification and Remote Diagnostics

**DOI:** 10.3390/bioengineering12020190

**Published:** 2025-02-16

**Authors:** Ying Fu, Shi Tan, Michel Kadoch, Jinghua Zhong, Lifeng Guo, Yangan Zhang, Xiaohong Huang, Xueguang Yuan

**Affiliations:** 1 Department of Ultrasound, Peking University Third Hospital, Beijing 100191, China; yingfu312@gmail.com (Y.F.); tanshi@gmail.com (S.T.); 2 Department of Electrical Engineering, École de Technologie Supérieure, Université du Québec, Québec, QC J5A 0M3, Canada; michel.kadoch@etsmtl.ca; 3 School of Electronic Engineering, Beijing University of Posts and Telecommunications, Beijing 100876, China; zhongjinghua@bupt.edu.cn (J.Z.); guolifeng@bupt.edu.cn (L.G.); zhang@bupt.edu.cn (Y.Z.); 4 School of Computer Science, Beijing University of Posts and Telecommunications, Beijing 100876, China; huangxh@bupt.edu.cn

**Keywords:** deep learning, medical image analysis, ultrasound imaging, lymph node classification, semantic-attention enhanced DSC-transformer

## Abstract

This study presents a novel Semantic-Attention Enhanced Dynamic Swin Convolutional Block Attention Module(CBAM) Transformer (DSC-Transformer) for lymph node ultrasound image classification. The model integrates semantic feature extraction and multi-scale attention mechanisms with the Swin Transformer architecture, enabling efficient processing of diagnostically significant regions while suppressing noise. Key innovations include semantic-driven preprocessing for localized diagnostic focus, adaptive compression for bandwidth-limited scenarios, and multi-scale attention modules for capturing both global anatomical context and local texture details. The model’s effectiveness is validated through comprehensive experiments on diverse datasets and Grad-Channel Attention Module (CAM) visualizations, demonstrating superior classification performance while maintaining high efficiency in remote diagnostic settings. This semantic-attention enhancement makes the DSC-Transformer particularly effective for telemedicine applications, representing a significant advancement in AI-driven medical image analysis with broad implications for telehealth deployment.

## 1. Introduction

Remote healthcare continues to evolve rapidly, driven by technological advances and increasing demand for accessible medical services. Digital technologies, including mobile health applications, wearable devices, and telemedicine platforms, have proven to be indispensable, particularly for patients in rural or underserved areas. This evolution brings unprecedented opportunities for expanding healthcare access while simultaneously presenting complex technical challenges. Traditional remote healthcare systems struggle with bandwidth limitations that affect the transmission of critical medical data, particularly high-resolution imaging studies essential for accurate diagnosis. These constraints become especially pronounced in regions lacking advanced infrastructure, where limited bandwidth can result in slow data transmission, poor quality, or data loss, all of which negatively affect the quality of care and patient outcomes.

Within this context, conventional communication approaches based on Shannon’s information theory increasingly show their limitations. While these traditional methods have served as the foundation for digital communication, they focus primarily on error-free transmission of raw data, leading to inefficiencies in medical data transmission. As healthcare systems increasingly rely on real-time data, there is a growing need for faster transmission speeds to support real-time diagnosis and decision-making. The large volume of medical data, often generated by diagnostic imaging devices and monitoring equipment, poses a significant burden on network resources.

Semantic communication emerges as a transformative solution by focusing on the meaning of the data being transmitted, ensuring that only the most relevant and critical information is sent. By focusing on the meaning and clinical significance of medical data rather than raw binary transmission, semantic communication systems can achieve substantially higher efficiency. A key principle is semantic feature extraction, which involves identifying the most important features within the data that are crucial for accurate diagnosis. These features are then compressed and transmitted, reducing data volume while preserving diagnostic value.

The application of semantic communication in lymph node ultrasound classification serves as a compelling case study for this technology’s potential. Lymph nodes, essential elements of the lymphatic system, play a vital role in immune response, acting as filters for harmful substances and providing early indicators of diseases such as cancer, infections, and autoimmune disorders. The accurate distinction between benign and malignant lymph nodes is critical in clinical settings, particularly for conditions like breast cancer, where early detection of metastasis can significantly impact prognosis and treatment outcomes. Existing classification systems have been developed to differentiate various lymph node abnormalities, such as necrotic changes, cystic formations, and calcifications. These features are indicative of non-malignant conditions, like infections or chronic inflammation, and must be carefully distinguished from malignancy. Such distinctions are essential for proper management and treatment decisions, as certain imaging features may overlap between benign and malignant nodes [1,2]. Traditional diagnostic methods, while effective, often rely heavily on clinician expertise, introducing subjectivity and potential errors [3,4].

Lymph node evaluation is not limited to ultrasound imaging; several other imaging modalities, such as computed tomography (CT) [5], magnetic resonance imaging (MRI) [6], positron emission tomography (PET) [7], and single-photon emission computed tomography (SPECT) [8], are commonly used in clinical practice. These modalities play critical roles in staging, treatment planning, and cases where ultrasound results are inconclusive. Additionally, elastography techniques, both ultrasound-based and MRI-based, are emerging tools for assessing tissue stiffness, which can help differentiate benign from malignant lymph nodes. Furthermore, lymphotropic contrast agents, which enhance lymph node visualization in ultrasound and MRI, have shown promise in improving diagnostic accuracy. While our study primarily focuses on ultrasound due to its accessibility and cost-effectiveness, the deep learning model proposed here can be adapted for use with other imaging modalities, provided that appropriate labeled data is available. An important factor in lymph node evaluation is size, as changes in size can indicate the effectiveness of therapy. Different anatomical sites may require distinct size thresholds and staging criteria, such as those outlined in the RECIST criteria for measuring target lesions, to assess treatment response accurately. Although our study primarily focuses on ultrasound due to its accessibility and cost-effectiveness, the deep learning model proposed here can be adapted to work with other imaging modalities, provided that appropriate labeled data is available.

Lymph node evaluation is crucial not only for assessing malignant tumors, including breast, pelvic, and head-and-neck cancers, but also for monitoring treatment response across various cancer types [9]. The role of lymph nodes extends beyond malignancies; they are also involved in non-malignant pathologies, such as sarcoidosis, tuberculosis, and Castleman disease. These non-malignant conditions can lead to changes in the appearance of lymph nodes, such as calcifications, cystic formations, or necrosis, which can sometimes be confused with malignancy. Differentiating these conditions is essential for accurate diagnosis and treatment planning. While the size of the lymph node is traditionally used in initial staging, it becomes especially important for tracking changes in response to treatment. For example, in pelvic-derived neoplasms, the size of the Lymph node is a key marker, with distinct criteria used compared to head and neck cancers. Different tumor sites may require unique size thresholds, such as those outlined by RECIST (Response Evaluation Criteria in Solid Tumors) for measuring target lesions during treatment assessment.

Recent advances in deep learning have revolutionized medical image analysis, offering automated and precise diagnostic tools. Zhang et al. provided a comprehensive review of deep learning applications in medical image analysis, emphasizing its crucial role in improving diagnostic accuracy and efficiency [10]. Liu et al. demonstrated that Convolutional Neural Networks (CNNs) established the initial benchmark for deep learning in medical imaging [11]. Wang et al. later introduced Vision Transformers (ViTs), showing superior performance in capturing global dependencies within medical images [12]. Hybrid CNN–Transformer models emerged, combining local feature extraction with long-range dependency modeling [13], while the integration of attention mechanisms enhanced classification performance [14]. However, challenges persist, particularly regarding limited labeled data and achieving consistent accuracy across different datasets and clinical settings [15,16].

The integration of Semantic Communications with deep learning-based lymph node classification methods opens new possibilities in telemedicine and IoT domains, particularly through enhanced semantic feature extraction and attention mechanisms. Through intelligent data transmission and semantic-aware information processing, this integration enhances the practicality and reliability of diagnostic systems, establishing a foundation for broader application scenarios. Addressing these opportunities and challenges, our study introduces the Semantic-Attention Enhanced Dynamic Swin–CBAM Transformer (DSC-Transformer) model, a novel approach that combines semantic feature extraction with advanced attention mechanisms. The proposed model builds upon the hierarchical structure of the Swin Transformer, incorporating semantic-driven preprocessing and multi-scale attention modules to prioritize diagnostically significant regions while suppressing noise in ultrasound images. The model’s architecture integrates the Convolutional Block Attention Module (CBAM) with dynamic convolution, enabling adaptive feature extraction and semantic compression for efficient data transmission in resource-constrained environments. This semantic-attention enhancement allows the model to adjust its operations based on specific input image characteristics, improving its ability to handle diverse features and patterns without significantly increasing model size, while maintaining high efficiency in data transmission and processing for remote healthcare applications.

The remainder of this paper is organized as follows. Section 2 discusses the principles of semantic communication and its applications in remote healthcare. Section 3 presents a detailed case study of lymph node ultrasound classification and remote diagnostics. Section 4 introduces our proposed Semantic-Attention Enhanced DSC-Transformer model, including its core architecture components and implementation details. Section 5 describes the implementation details and training methodology, covering dataset preparation, data augmentation, training strategies, and validation approaches. Section 6 presents comprehensive experimental results and analysis, comparing our model’s performance against state-of-the-art classification models and conducting ablation studies. Section 7 discusses the strengths, limitations, and future directions of our approach. Finally, Section 8 concludes this paper.

## 2. Semantic Communication in Remote Healthcare

### 2.1. Principles of Semantic Communication

Semantic communication is a paradigm shift from traditional communication systems, focusing not just on transmitting raw data but on delivering meaningful, context-aware information. This approach is particularly advantageous in healthcare, where the goal is not just to send large volumes of data but to transmit information that is crucial for timely diagnosis and decision-making. In bandwidth-limited scenarios of remote telemedicine consultations or mobile networks, semantic communication systems can automatically adjust data transmission based on network conditions. The system prioritizes critical diagnostic data when bandwidth is low. This includes information about tumors, abnormal growths, or suspicious organ changes. Meanwhile, less important data like background noise or non-diagnostic regions undergo compression or omission. This ensures that the most urgent and clinically relevant information reaches the healthcare provider with minimal delay, improving the timeliness and effectiveness of the diagnosis.

**Figure 1:**
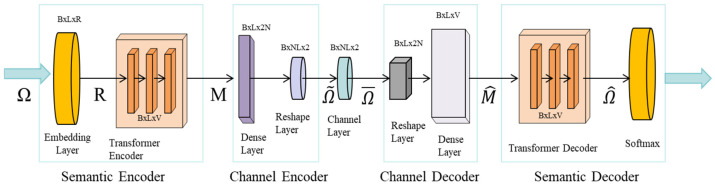
The architecture of the semantic encoder/decoder and channel encoder/decoder models of the proposed SemCom system model.

Moreover, the ability of semantic communication to adapt based on clinical priorities introduces a level of personalization to the transmission process. For instance, a system can prioritize certain types of data based on the urgency of the clinical context. In emergency situations, where time is of the essence, the system can prioritize high-risk information, such as the presence of cancerous lesions, or clinical signs of acute conditions, like heart attacks or strokes, over routine or non-urgent data. This ensures that clinicians receive the most relevant information first, allowing for faster decision-making, which can be life-saving in critical care scenarios. Once critical features have been identified, semantic compression plays a pivotal role in reducing the volume of data that needs to be transmitted without compromising the essential diagnostic content. The image displayed in Figure 1 illustrates how a medical image can be compressed by focusing on the clinically relevant regions, leaving out areas that do not contribute to the diagnosis. This method significantly reduces the amount of data required for transmission, ensuring faster delivery without losing important diagnostic details.

The first step in semantic communication is extracting meaningful information from raw data. In the context of medical imaging, for instance, this could involve identifying key features in ultrasound or CT scans that are indicative of a particular condition. Instead of transmitting the entire image, semantic communication identifies and focuses on the most diagnostically relevant features, such as tumors or lesions in the case of cancer detection.

Once critical features have been identified, semantic compression reduces the volume of the data needed to be transmitted without losing essential diagnostic content. By focusing only on relevant aspects of the data, this method significantly reduces the amount of bandwidth required. For example, a detailed medical image may be compressed by discarding unnecessary regions of the image while preserving the parts that contribute to clinical decision-making. In a semantic communication system, we can define the Semantic Extraction (SE) efficiency $\eta_{s}$ as

(1) $$\eta_{s}=S_{m}/S_{t}$$

where $S_{m}$ is the amount of meaningful semantic information extracted, and $S_{t}$ is the total amount of original data.

**Figure 2:**
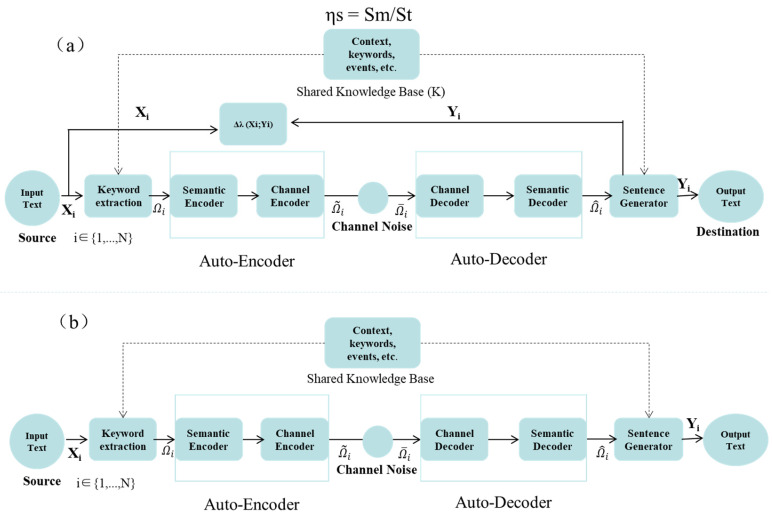
The block diagram of our proposed SemCom system model. The model in (**a**) is used for training the system parameters and the model in (**b**) is used for evaluating the SemCom system.

Moreover, the transmission strategy can be dynamically adapted based on network conditions. Figure 2 provides an example of how this adaptation works, showing the process of prioritizing critical diagnostic data when bandwidth is limited. During low-bandwidth periods, regions of the image that are most relevant for the diagnosis, such as the central area showing a potential malignancy, are transmitted first, ensuring that clinicians have access to the most urgent information as soon as possible. One of the core features of semantic communication is its ability to adapt the transmission strategy based on network conditions and clinical priorities. In a bandwidth-constrained environment, the system can dynamically adjust the amount of data being transmitted. For example, during periods of low bandwidth, the system could prioritize critical diagnostic data, ensuring that the most urgent information reaches the clinician in real-time. For medical image compression, the compression ratio R can be expressed as

(2) R=Do/Dc

where Do is the original data size, and Dc is the compressed data size.

In remote healthcare scenarios, the end-to-end delay T of semantic communication can be represented as

(3) $$T=T_{se}+T_{t}+T_{sd}$$

where $T_{se}$ is semantic encoding time, $T_{t}$ is transmission time, and $T_{sd}$ is semantic decoding time.

Looking ahead, the integration of semantic communication with machine learning (ML) technologies could further enhance its capabilities. AI algorithms could be used to automatically identify and classify relevant features in medical images, enabling even more precise compression and data prioritization. Machine learning models could also be used to predict the most critical data that need to be transmitted based on the patient’s history, the current clinical context, and the urgency of the diagnosis. This level of automation would allow healthcare providers to focus more on patient care, while the system handles the complexity of managing data transmission efficiently and effectively.

In conclusion, semantic compression and communication represent a significant leap forward in medical image transmission, offering the potential to enhance diagnostic accuracy, reduce bandwidth consumption, and ensure that critical information is delivered to healthcare professionals in real-time. By adapting to network conditions and clinical priorities, this approach ensures that the most relevant diagnostic content reaches clinicians promptly, improving patient outcomes and the overall efficiency of healthcare delivery. As the field of medical imaging continues to evolve, the integration of semantic communication will play a crucial role in overcoming the challenges posed by bandwidth limitations, ensuring that remote healthcare and telemedicine can continue to thrive.

### 2.2. Advantages for Remote Healthcare

Semantic communication presents numerous advantages for remote healthcare systems, where the efficient transmission of medical data is vital for timely decision-making and intervention.

In remote healthcare environments, where network resources may be limited, semantic communication ensures that only essential data is transmitted. By focusing on diagnostically critical information, it significantly reduces the volume of data being sent without compromising the quality or accuracy of the diagnosis. This is particularly important in telemedicine, where bandwidth constraints are common in rural or underdeveloped areas.

With semantic communication, healthcare professionals can receive faster data transmission and processing, which is crucial for real-time diagnosis and treatment decisions. The reduced data volume allows for quicker analysis, enabling healthcare providers to act swiftly and improve patient outcomes. For instance, when diagnosing a critical condition, such as stroke or sepsis, the ability to rapidly transmit key medical data can make a significant difference in treatment success.

Semantic communication improves the explainability of transmitted data. By focusing on the most relevant diagnostic content, the system presents clinicians with clearer, more interpretable information. This can foster greater trust and confidence in the system, especially when clinicians rely on machine-learning models for decision support. The ability to explain why certain information was prioritized helps clinicians understand the reasoning behind automated suggestions, ensuring they can make informed decisions.

### 2.3. Integration with IoT and Telemedicine

The integration of semantic communication with IoT-enabled medical devices opens up new possibilities for continuous monitoring and diagnostics in remote healthcare.

Many healthcare devices, such as wearable sensors, are now capable of continuously collecting patient data. In remote healthcare, where patients may not be physically present in a clinic or hospital, these devices allow for constant monitoring of vital signs, like heart rate, blood pressure, and oxygen levels. Semantic communication helps by efficiently transmitting only the most relevant data, such as critical changes in a patient’s condition, rather than sending large volumes of routine measurements. This ensures that healthcare professionals receive timely updates while minimizing bandwidth use.

In telemedicine, where remote consultations between patients and healthcare providers are becoming more common, semantic communication enables real-time exchanges of essential clinical data. Even in bandwidth-limited environments, semantic communication ensures that clinicians can share meaningful data, such as images, test results, and diagnostic information, with experts remotely. This makes it possible for healthcare providers to receive support from specialists without requiring high-bandwidth connections. Whether in an emergency situation requiring immediate consultation or a routine check-up, semantic communication ensures that vital information is shared effectively.

By leveraging semantic communication, IoT-enabled devices, and telemedicine, remote healthcare systems can offer enhanced patient care, particularly in underserved regions with limited resources. The intelligent transmission of relevant data enables faster diagnosis, better decision-making, and more efficient resource utilization.

## 3. Lymph Node Ultrasound Classification and Remote Diagnostics: A Case Study

### 3.1. Clinical Importance of Lymph Node Classification

Lymph nodes are critical immune system structures that serve as filtration points for harmful substances, including pathogens, cancer cells, and other potential threats. Their role extends far beyond just immune defense; they are also key indicators in diagnosing a wide range of conditions, including infections, autoimmune diseases and, most notably, cancers. Lymph node classification, particularly in imaging, is an essential part of clinical decision-making. It allows healthcare providers to identify whether a lymph node is benign or malignant, which has profound implications for diagnosis and treatment planning.

The clinical importance of lymph node classification is particularly pronounced in the context of oncology. Lymph node metastasis often represents a critical stage in cancer progression, as it indicates the spread of cancer cells from the primary tumor site to distant regions of the body. In cancers such as breast cancer, cervical cancer, lung cancer, and melanoma, the involvement of regional lymph nodes significantly affects the staging of the disease, which, in turn, guides therapeutic strategies. For instance, breast cancer staging relies heavily on lymph node involvement to determine the extent of the disease and whether the cancer has spread beyond the breast tissue.

The timely detection of malignancy in lymph nodes can directly influence patient outcomes. In breast cancer, for example, identifying metastatic lymph nodes early can enable more accurate staging and treatment decisions. If detected early, the patient can undergo less invasive procedures, such as targeted surgery or localized radiation therapy. On the other hand, if lymph node metastasis is not identified in a timely manner, patients may undergo aggressive treatments that could have been avoided, or the cancer may spread to other parts of the body, making treatment less effective.

Early detection of lymph node abnormalities also extends beyond cancer diagnosis. In the case of infections and autoimmune diseases, lymph node enlargement or abnormal morphology can be indicative of an ongoing pathological process. For instance, in diseases like tuberculosis, and even systemic lupus erythematosus (SLE), the lymph nodes may present with characteristic changes that help clinicians distinguish between these conditions and other causes of lymphadenopathy. Timely diagnosis in these cases can significantly alter the treatment plan and reduce the risk of complications associated with misdiagnosis.

Thus, the ability to classify lymph nodes accurately in imaging is essential not only for identifying cancers but also for detecting infections and autoimmune diseases early, ultimately contributing to better patient care, more efficient use of healthcare resources, and improved prognosis.

### 3.2. Challenges in Remote Diagnostics for Lymph Nodes

While lymph node classification is vital for effective clinical decision-making, several challenges complicate accurate diagnosis, particularly in remote diagnostic settings. One of the foremost challenges is the limited availability of labeled datasets for training machine learning and deep learning models. Medical imaging datasets, especially those with expert annotations, are scarce and difficult to obtain. Expert radiologists, oncologists, or pathologists must manually annotate the images, which is a time-consuming and costly process. Moreover, the scarcity of labeled data is particularly problematic when dealing with rare conditions, variations in disease progression, or atypical cases. This lack of sufficient training data makes it difficult to build robust deep learning models that can generalize across various types of patients, conditions, and imaging devices.

Another significant challenge in remote diagnostics for lymph node classification is the variability and noise present in ultrasound images. Ultrasound imaging, while non-invasive and widely accessible, is highly susceptible to image artifacts and noise due to factors like operator experience, patient movement, and body composition. For example, in lymph node ultrasound imaging, distinguishing benign from malignant nodes can be challenging when the images are distorted by artifacts such as speckle noise, shadowing, or motion blurring. Additionally, the appearance of lymph nodes can vary greatly depending on their location, size, shape, and surrounding tissue. This variability adds another layer of complexity to the task of classifying these nodes accurately.

The high bandwidth requirements for transmitting high-resolution ultrasound images in real-time also present a significant challenge. In remote or rural healthcare settings, where internet bandwidth may be limited or unreliable, transmitting large medical images in real-time can be problematic. Ultrasound images, particularly those with high resolution necessary for detailed classification, often contain a large amount of data. Transmitting this data over low-bandwidth networks can result in delays, poor-quality images, or incomplete transmissions, all of which hinder the ability of clinicians to make timely and accurate decisions. These transmission issues are especially critical in emergency situations, where every minute counts in diagnosing and treating conditions like cancer or infections.

Lastly, ensuring that the data received by clinicians is both accurate and up-to-date presents additional challenges. Remote diagnostics rely heavily on timely communication but delays in data transmission can lead to discrepancies between the actual clinical situation and what the clinician is reviewing. These delays can affect decision-making, especially if the transmitted images do not accurately reflect the current state of the patient.

### 3.3. Semantic Communication as a Solution

Semantic communication offers a promising solution to these challenges by optimizing the way data is transmitted and processed in medical imaging, particularly in remote diagnostics for lymph node classification. This advanced communication model focuses on transmitting only the most diagnostically relevant information while suppressing unnecessary or redundant data. By doing so, semantic communication significantly reduces the amount of data required for transmission, alleviating the bandwidth burden typically associated with high-resolution medical images.

The key advantage of semantic communication lies in its ability to enhance the efficiency of data transmission. In the context of lymph node ultrasound images, the system can be designed to prioritize the most clinically significant regions of the image—those that show potential abnormalities such as enlarged nodes, irregular shapes, or unusual tissue patterns. For instance, if a particular area of the lymph node shows signs of malignancy, semantic communication would transmit high-resolution data from this region while compressing or discarding other less critical areas of the image, such as the surrounding healthy tissue. This process reduces the total volume of data transmitted, making it possible to share real-time images even in bandwidth-limited environments.

Furthermore, semantic communication leverages intelligent data transmission that adapts to network conditions in real time. When bandwidth is constrained, the system can dynamically adjust the amount of data transmitted, ensuring that critical diagnostic information reaches clinicians with minimal delay. For example, in an emergency situation where a clinician needs immediate information about a potentially cancerous lymph node, the system can prioritize the transmission of the region most likely indicating malignancy, ensuring that the clinician receives the most relevant data first. Once the critical information has been transmitted, the system can send additional data if needed, allowing clinicians to access more detailed images when the network permits.

In addition to improving the speed and efficiency of data transmission, semantic communication can support clinician decision-making by highlighting critical features in the image through advanced feature extraction techniques. These techniques use machine learning algorithms to identify and emphasize key regions within the image that may require immediate clinical attention. By focusing on areas that are most relevant to the diagnosis, semantic communication not only enhances the efficiency of data transmission but also supports more accurate and timely decision-making. This is particularly important in remote settings, where clinicians may have limited access to resources and need to rely on technology to assist in making quick and accurate diagnoses.

Ultimately, the integration of semantic communication into remote diagnostics can revolutionize the way healthcare providers diagnose and treat conditions involving lymph node abnormalities. By improving the speed and efficiency of data transmission, reducing bandwidth usage, and enhancing feature extraction, semantic communication ensures that clinicians have access to the most relevant diagnostic information when and where it is most needed. This can significantly improve outcomes for patients, particularly in remote or underserved areas where timely access to specialized care is often limited.

## 4. Proposed Model: Semantic-Attention Enhanced DSC-Transformer

### 4.1. Overview of the DSC-Transformer

The Dynamic DSC-Transformer advances medical image analysis through two key innovations: semantic-driven preprocessing and multi-scale attention mechanisms built upon the Swin Transformer architecture. At its core, the model integrates the CBAM for enhanced feature extraction and dynamic convolution for adaptive processing.

The channel attention mechanism focuses on the inter-channel dependencies of the feature maps. It can be described as

(4) $$M_{c}\left(F\right)=\sigma \left(\mathrm{MLP}\left(\mathrm{AvgPool}\left(F\right)\right)+\mathrm{MLP}\left(\mathrm{MaxPool}\left(F\right)\right)\right)$$

where $F\in {\mathbb{R}}^{H\times W\times C}$ is the input feature map, AvgPool and MaxPool are global average and max pooling operations applied along the spatial dimensions, and σ is the sigmoid activation function.

The output is a set of attention weights applied to each channel:

(5) $$F^{\prime }=M_{c}\left(F\right)\cdot F$$

where $M_{c}\left(F\right)\in {\mathbb{R}}^{1\times 1\times C}$ contains the attention weights, and ⋅ represents element-wise multiplication.

This dual-layer attention allows the model to prioritize the most important feature channels and focus on the spatial regions that are most relevant, such as subtle differences in lymph node textures that may indicate malignancy. This improves the model’s ability to highlight diagnostically critical areas effectively.

In parallel, dynamic convolution operates by generating kernel weights based on the input characteristics, which can be mathematically expressed as

(6) $$W_{\mathrm{dyn}}\left(x\right)=g\left(x;\theta \right)$$

where $W_{\mathrm{dyn}}$ represents the dynamically generated kernel weights, x is the input, and $g\left(x;\theta \right)$ is a learnable function (typically a small neural network) that generates the weights. Unlike static convolutions, where the same weights are used for all inputs, dynamic convolution adapts to each image.

The output of dynamic convolution can be formulated as

(7) $$y=W_{\mathrm{dyn}}\left(x\right)*x$$

where * represents the convolution operation. This adaptability allows the model to capture varying patterns in lymph node images more effectively.

Unlike static convolutions that apply the same filter to all images, dynamic convolution tailors its operations to the specific visual patterns of each ultrasound image, capturing both fine-grained local details and broader global structures [17]. This adaptability is particularly beneficial in medical imaging, where subtle variances between benign and malignant tissue must be discerned without adding unnecessary computational complexity.

The Swin Transformer is known for its hierarchical design and efficient feature extraction capabilities. The addition of CBAM enhances the model’s ability to focus on relevant features, while dynamic convolution allows the model to adaptively adjust its operations based on input characteristics [18]. This integration significantly improves the model’s accuracy and adaptability in lymph node ultrasound image classification, making the Dynamic DSC-Transformer particularly well-suited for this complex medical image analysis task.

The multi-scale attention mechanism is realized through the hierarchical design of the Swin Transformer, enhanced by dynamic convolution capabilities. Unlike traditional approaches, this combination enables adaptive processing across different spatial scales, crucial for analyzing medical images where features of interest may appear at various resolutions [19,20]. The dynamic convolution component automatically adjusts its operations based on input characteristics, allowing for precise capture of both local details and global structures without increasing computational complexity.

The integration of these components—semantic preprocessing, CBAM attention, and multi-scale feature extraction—creates a robust framework specifically optimized for medical image analysis tasks, such as lymph node classification.

### 4.2. Core Architecture Components

(a). Patch Segmentation Module

**Figure 3:**
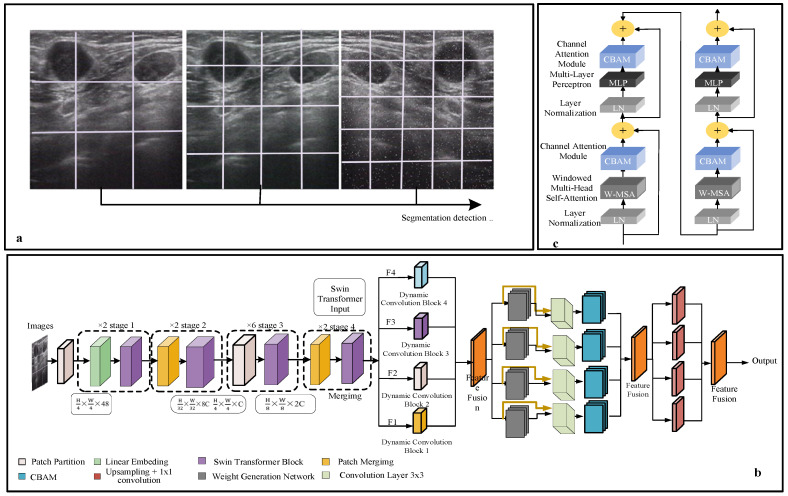
DSC-Transformer model architecture: (**a**) Multi-scale segmentation of ultrasound images, demonstrating the model’s hierarchical feature extraction capability. (**b**) Overall network structure, including patch partitioning, linear embedding, multi-stage Swin Transformer blocks, dynamic convolution blocks, and feature pyramid structure. (**c**) Internal structure of two consecutive Swin Transformer blocks, integrating CBAM.

The initial phase of our architecture is the Patch Segmentation [21] Module, depicted in Figure 3a. This module divides the input ultrasound image into smaller, non-overlapping patches through a patch extraction process, represented mathematically by the following equation:

(8) $$I=U_{i-1}^{N}P_{i}$$

(9) $$P_{i}\cap P_{j}=\emptyset \forall i\neq j$$

where I denotes the input image, and $P_{i}$ are the non-overlapping patches, such that their union reconstitutes the original image with no overlap. Each patch captures a localized region of the image, enabling the model to process and analyze granular details essential for high-resolution medical imaging.

Post-segmentation, each patch $P_{i}$ is transformed into a high-dimensional feature space through a linear embedding layer E, followed by a normalization process to stabilize the training dynamics:

(10) $$\begin{array}{c} z_{i}^{0}=LayerNorm\left(E\cdot vec\left(P_{i}\right)\right) \end{array}$$

where $vec\left(•\right)$ denotes the operation of flattening the patch pixels into a vector, and E is a trainable embedding matrix. Layer normalization is applied to each embedded patch vector to ensure consistent scale across different features.

Following patch segmentation and embedding, the Downsampling Module aggregates the information from multiple embedded patches. This module performs a dimensionality reduction operation through learned transformations, which merge adjacent embedded patches into a single, lower-resolution but higher-dimensional representation:

(11) $$\begin{array}{c} x_{k}^{\prime }=Reduce\left(\begin{array}{c} \underset{j\in 5_{i}}{⊕z_{j}^{0}} \end{array}\right) \end{array}$$

where ⊕ denotes a concatenation operation over the set $S_{k}$ of indices of the patches being merged, and Reduce(•) is a transformation that combines these concatenated features into a single vector with reduced spatial dimensions but expanded feature dimensions, typically implemented through a convolution or a dense layer with non-linearity. Here is the updated section with the specific changes applied to the formulas:

(12) $$\begin{array}{c} u^{l+1}=\mathrm{TransformerBlock}(u^{l}) \end{array}$$

(13) $$\begin{array}{c} T\left(u^{l}\right)=MSA\left(LN\left(u\right)\right)+u\left(14\right)=MLP\left(LN\left(u\right)\right)+u \end{array}$$

The Swin Transformer model integrates multi-head self-attention (MSA), multilayer perceptrons (MLP), and layer normalization (LN) to process and analyze complex image data effectively. The core component, MSA, dynamically assigns importance to different parts of an image, refining the features extracted from each patch and focusing on relevant patterns while reducing noise. In medical imaging, such as lymph node ultrasound analysis, this dynamic weighting is crucial for prioritizing regions of interest that may indicate disease, allowing the model to focus on areas that are most clinically significant while disregarding irrelevant regions.

The hierarchical feature extraction process begins by dividing the input image into smaller patches, applying the self-attention mechanism to capture both local details and broader global patterns. This is particularly important in medical contexts, where small anomalies can be embedded within larger structures. By processing the image in patches, the model can maintain a high degree of precision when identifying subtle differences in tissue morphology, such as irregularities in lymph nodes that might indicate malignancy, enabling a more accurate diagnostic outcome.

In addition to MSA, the model incorporates MLPs to further refine features and improve the ability to differentiate between benign and malignant characteristics. LN stabilizes the training process by normalizing inputs, preventing issues like vanishing or exploding gradients, ensuring the model’s robustness across a wide range of medical images. The Swin Transformer strikes a balance between computational efficiency and performance, making it well-suited for medical imaging tasks that require both local detail processing and contextual integration for accurate diagnosis, as demonstrated in the analysis of lymph node ultrasound images.

(b). Swin Transformer Block

The core advantage of the Swin Transformer in our study is reflected in its unique self-attention computational method, as shown in Figure 3b. Combined with Dynamic Convolution, this structure further enhances the model’s accuracy in classifying lymph node ultrasound images [22]. The Swin Transformer’s hierarchical design implements semantic-aware processing through its unique self-attention computation and moving window strategy. Operating across four stages with progressively reduced spatial resolution but expanded feature dimensions, it efficiently captures both local details and global patterns in lymph node images.

This architecture aligns with semantic communication goals by optimizing computational efficiency through non-overlapping windows while maintaining cross-window connections, enabling effective processing of high-resolution medical images. The window-based attention mechanism, governed by Equations (11)–(22), strategically balances local feature preservation with global context understanding, making it particularly effective for detecting subtle diagnostic patterns while maintaining computational efficiency through its multi-head attention design and shifting window approach.

In each Swin Transformer block, the self-attention computation is performed as follows:

(14) $$\begin{array}{c} Attention\left(Q,K,V\right)=SoftMax\left(\frac{QK^{T}}{\sqrt{d}}+B\right)V \end{array}$$

where $Q,K,V\in {\mathbb{R}}^{\left(M^{2}\times d\right)}$ represent the query, key, and value matrices respectively, d is the dimensionality, $M^{2}$ is the number of blocks in the window, and $B\in {\mathbb{R}}^{\left(M^{2}\times M^{2}\right)}$ is the relative positional bias, which is crucial for model performance.

The Swin Transformer adopts a moving window partition strategy, alternating between different window configurations between consecutive self-attention layers.

In multi-head self-attention, the attention mechanism is applied multiple times in parallel, using different learned linear projections for each head. For a single head, the attention mechanism is computed as:

(15) $$Attention\left(Q,K,V\right)=SoftMax\left(\frac{QK^{⊤}}{\sqrt{d_{k}}}\right)V$$

In multi-head attention, this process is repeated h times (the number of heads), each with its own learned projections $W_{Q}^{i},W_{K}^{i},W_{V}^{i}$. The output of each head is concatenated and linearly transformed:

(16) $$Multihead\left(Q,K,V\right)=Concat\left(head_{1},\ldots ,head_{h}\right)W_{O}$$

where each $head_{i}=Attention\left(QW_{Q}^{i},KW_{K}^{i},VW_{V}^{i}\right)$, and $W_{O}$ is a learned weight matrix used to combine the outputs of all heads.

This strategy allows for cross-window connections while maintaining the computational efficiency of nonoverlapping windows. The specific computational process is as follows:

(17) $$u^{l}=W-MSA\left(LN\left(u^{l-1}\right)\right)+u^{l-1}$$

(18) $$\;u^{l}=MLP\left(LN\left(u^{l}\right)\right)+u^{l}$$

(19) $$u^{l+1}=SW-MSA\left(LN\left(u^{l}\right)\right)+u^{l}$$

(20) $$\begin{array}{c} \;u^{l+1}=MLP\left(LN\left(u^{l+1}\right)\right)+u^{l+1} \end{array}$$

where W−MSA and SW−MSA, respectively, represent the regular and shifting window MSA modules, and LN denotes layer normalization.

The Swin Transformer features a hierarchical structure, which gradually reduces the resolution of feature maps by merging features from adjacent spatial blocks. For an input image of size H×W, the resolutions of the feature maps at different stages are as follows:

Stage 1:

(21) $$\frac{H}{4}\times \frac{W}{4}$$

Stage 2:

(22) $$\frac{H}{8}\times \frac{W}{8}$$

Stage 3:

(23) $$\frac{H}{16}\times \frac{W}{16}$$

Stage 4:

(24) $$\begin{array}{c} \;\frac{H}{32}\times \frac{W}{32} \end{array}$$

This hierarchical design enables the Swin Transformer to efficiently process visual features at different scales, similar to traditional CNNs, while maintaining efficient computation for high-resolution images. The complexity of the self-attention computation in Swin Transformer is

(25) $$\begin{array}{c} \Omega \left(MSA\right)=4hwC^{2}+2M^{2}hwC \end{array}$$

where h and w are the height and width of the feature map, C is the number of channels, and M is the window size.

(c). Dynamic Convolution

A key addition to our architecture is the Dynamic Convolution module. Unlike standard convolutions where kernel weights remain fixed after training, dynamic convolution generates kernel weights on-the-fly based on the input. The dynamic convolution operation can be formulated as

(26) $$\begin{array}{c} y=f\left(x,W\left(x\right)\right) \end{array}$$

where x is the input, $W\left(x\right)$ is the dynamically generated weight, and $f\left(•\right)$ is the convolution operation. The weight generation function $W\left(x\right)$ is typically implemented as a small neural network:

(27) $$\begin{array}{c} W\left(x\right)=g\left(x,\theta \right) \end{array}$$

where $g\left(•\right)$ is the weight generation network with parameter θ. The output of this weight generation network is then used to parameterize the main convolution operation. In practice, we can express the dynamic convolution as a sum of basis convolutions:

(28) $$\begin{array}{c} y=\sum_{k=1}^{K}\pi_{k}\left(x\right)*\left(W_{k}*x\right) \end{array}$$

where $\pi_{k}\left(x\right)$ are input-dependent mixing coefficients, $W_{k}$ are learnable basis filters, and * denotes the convolution operation. The mixing coefficients are computed by the weight generation network:

(29) $$\begin{array}{c} \pi \left(x\right)=softmax\left(h\left(x\right)\right) \end{array}$$

where $h\left(x\right)$ is another small neural network. This formulation allows the model to adaptively combine different convolution kernels based on the input, enhancing its flexibility and expressiveness.

The computational complexity of dynamic convolution can be expressed as

(30) $$\begin{array}{c} \Omega \left(DynConv\right)=hwC\left(K+C_{in}\right) \end{array}$$

where h and w are the height and width of the feature map, C is the number of output channels, K is the number of basis filters, and $C_{in}$ is the number of input channels.

The integration of dynamic convolution into our Swin Transformer model enables adaptive feature extraction based on each lymph node image’s unique characteristics. Unlike static convolutions, dynamic convolution generates filter weights on-the-fly, handling diverse inputs efficiently without increasing model complexity. By combining the Swin Transformer’s hierarchical structure for multi-scale feature capture with dynamic convolution’s adaptive capabilities, our model effectively distinguishes between benign and malignant lymph nodes through analysis of both fine details and larger structures. This integration improves classification accuracy and adaptability while maintaining computational efficiency, making it a valuable tool for clinical diagnosis and improving patient outcomes.

### 4.3. Semantic Feature Extraction and Compression

To enhance the efficiency of remote diagnostic systems, our model incorporates semantic compression and feature extraction techniques that play a vital role in optimizing network communications, particularly crucial for IoT and telemedicine applications where bandwidth resources are often constrained. These techniques enable the model to minimize data transmission volume while preserving essential diagnostic information from lymph node ultrasound images.

The semantic feature extraction and compression in our model is implemented through a hierarchical structure, as illustrated in Figure 3. The process begins with multi-scale segmentation (Figure 3a), where ultrasound images are divided into patches at different scales, enabling the model to capture semantic information at various granularities.

In the overall network architecture (Figure 3b), semantic feature extraction and compression progress through a hierarchical structure that systematically reduces spatial dimensions while enriching feature representations. Starting from Stage 1 (H × W × 48) with initial feature extraction through patch partitioning and linear embedding, the architecture progresses through Stage 2 (H/2 × W/2 × C) for first-level compression and feature refinement. It then advances to Stage 3 (H/4 × W/4 × 2C) for further feature consolidation and concludes at Stage 4 (H/8 × W/8 × 2C) with final compression and high-level feature extraction. This progressive strategy ensures the preservation of crucial semantic information while reducing data dimensionality.

Each stage integrates Swin Transformer blocks with CBAM (Figure 3c), where the attention mechanisms help identify and prioritize diagnostically relevant features. The gradual reduction in spatial dimensions (H × W → H/8 × W/8) coupled with the increase in feature channels (48 → 2C) represents our semantic compression strategy, ensuring that essential diagnostic information is preserved while reducing data dimensionality.

The semantic feature extraction process focuses on identifying diagnostically significant regions within lymph node ultrasound images. This can be mathematically expressed through the feature extraction function:

(31) f(x)=Φ(Wx+b)

where x represents the input ultrasound image data, W and b are the weight and bias parameters of the feature extraction network, and Φ(•) is the activation function (typically ReLU or Sigmoid). This function processes the input data to extract key features that are most relevant for lymph node classification.

For data compression, we employ the Variational Information Bottleneck (VIB) method, which effectively balances information preservation with data reduction. The VIB objective function is formulated as

(32) $$LVIB\;=E_{q(z|x)}\;[logp(y|z)]-\beta \cdot KL(q(z|x)∥p(z))$$

where q(z|x) represents the posterior distribution of compressed features, p(z) is the prior distribution, and p(y|z) denotes the probability distribution of the classification target given the compressed features. The hyperparameter β balances reconstruction accuracy with compression rate, while KL represents the Kullback–Leibler divergence, measuring the difference between posterior and prior distributions.

The first term $E_{q(z|x)}\;[logp(y|z)]$ ensures that the compressed representation maintains high classification accuracy, while the second term KL(q(z|x)∥p(z)) promotes efficient compression by reducing redundant information. This approach allows our model to achieve significant data reduction while preserving the critical features necessary for accurate lymph node classification.

The synergy between semantic feature extraction and compression enhances the overall diagnostic process. The feature extraction mechanism identifies and highlights regions that are most indicative of lymph node abnormalities, while the compression algorithm efficiently condenses this information for transmission. This combination optimizes bandwidth usage while maintaining diagnostic accuracy, enabling real-time or near-real-time classification in bandwidth-constrained environments. The approach is particularly valuable in remote healthcare settings, where efficient data transmission is essential for timely diagnosis and treatment decisions.

### 4.4. Integration of CBAM and Dynamic Convolution

The model architecture of the Dynamic DSC-Transformer significantly enhances the Swin Transformer by integrating two key innovations: the CBAM and dynamic convolution, both of which play crucial roles in improving the classification of lymph node ultrasound images. These additions bolster the model’s ability to focus on the most relevant features of medical images while adapting to the varying characteristics of each input. Figure 3c provides a visual depiction of these enhancements to the Swin Transformer Block, showcasing how the incorporation of CBAM and dynamic convolution refines the model’s performance by focusing attention on critical areas and dynamically adjusting its convolution operations [23].

The Dynamic DSC-Transformer’s architecture begins with the Patch Segmentation Module, a key component that divides the input ultrasound image into smaller, non-overlapping patches. This patch-based approach allows the model to focus on capturing intricate local details that are essential for high-resolution medical image analysis. Each patch contains a localized section of the image, enabling the model to process smaller portions of the image independently, which is especially useful in medical contexts where subtle variations in tissue or structure can signal important diagnostic information.

Following the patch segmentation process, the model utilizes a Downsampling Module, which aggregates and merges these patches to reduce computational complexity without losing critical information about the overall structure of the image. This hierarchical approach allows the model to maintain a balance between focusing on fine-grained details at the local level and preserving the broader context of the entire image. The downsampling process reduces the spatial resolution of the image while expanding the feature space, enabling the model to retain essential features even as it simplifies the data for further processing.

By integrating the strengths of CBAM and dynamic convolution, the Dynamic DSC-Transformer not only captures and emphasizes clinically significant regions within the image but also adapts its operations based on the unique properties of each image. This adaptability and attention-driven focus make it particularly well-suited for the complex task of classifying lymph node ultrasound images, where both fine local details and broader structural patterns must be analyzed to differentiate between benign and malignant nodes. Ultimately, the enhanced architecture of the Dynamic DSC-Transformer leads to superior classification performance and greater clinical utility.

At the core of the model, the Enhanced Swin Transformer Block utilizes self-attention mechanisms and feed-forward neural networks to process the downsampled patches, effectively capturing both local and global features. The integration of dynamic convolution allows the model to adaptively adjust its operations based on input-specific characteristics, enhancing its ability to handle diverse features and patterns without significantly increasing model size.

CBAM significantly enhances the feature extraction capabilities of the Dynamic DSC-Transformer by introducing dual attention mechanisms—channel attention and spatial attention. These attention mechanisms play a vital role in guiding the model to focus on the most diagnostically relevant features of lymph node ultrasound images, which in turn improves the model’s classification accuracy. The CBAM module operates by sequentially applying these two types of attention, ensuring that both the feature channels and the spatial regions within the image that are most likely to contain critical information receive the most emphasis.

Channel attention refines the model’s focus by weighing the importance of different feature channels. In medical images, like those of lymph nodes, certain feature channels may capture textures, edges, or patterns that are more indicative of malignancy or other pathological conditions. By emphasizing these important channels, the model becomes more sensitive to the features that contribute most significantly to accurate diagnoses. The channel attention mechanism effectively filters out less important features and concentrates computational resources on the channels that provide the most diagnostic value, thereby enhancing the model’s ability to detect subtle signs of disease.

Spatial attention, on the other hand, directs the model’s focus to critical areas within the image itself. In ultrasound images, where abnormalities in lymph node morphology can be small or difficult to detect, spatial attention helps the model prioritize regions of interest, such as irregular tissue structures or unusual shapes that might indicate malignancy. This mechanism ensures that the model does not waste attention on irrelevant background areas, instead honing in on the portions of the image that are most likely to contain meaningful clinical information. Spatial attention is especially useful in ensuring that the model’s classification decisions are based on clinically significant areas, which leads to more reliable diagnostic outcomes.

Together, these attention mechanisms within the CBAM create a synergistic effect, allowing the model to be highly focused both in terms of the features it extracts and the regions it analyzes. This integrated approach—combining the hierarchical structure of the Swin Transformer, the adaptive processing power of dynamic convolution, and the precise focus provided by CBAM—ensures that the model efficiently processes and accurately classifies lymph node ultrasound images. By concentrating on the most relevant aspects of the image data, the model is able to deliver superior classification results, making it a valuable tool in clinical settings where accurate and timely diagnosis is crucial. Ultimately, the incorporation of CBAM not only boosts the overall performance of the model but also contributes to its ability to assist clinicians in making more informed and accurate diagnostic decisions.

**Figure 4:**
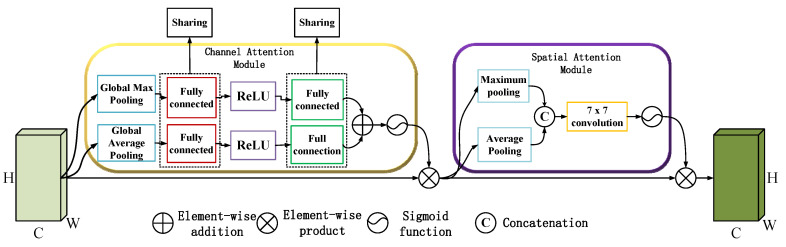
Architecture of the CBAM.

The CBAM module plays a pivotal role in our model by introducing a dual attention mechanism that enhances feature extraction [24]. In Figure 4, it comprises two sub-modules: the Channel Attention Module (CAM) and the Spatial Attention Module (SAM).

CAM: This sub-module focuses on the inter-channel relationships of the feature maps. It applies a squeeze-and-excitation operation to emphasize important channels, thereby improving the model’s ability to recognize relevant features across different channels:

(33) $$\begin{array}{c} N_{c}\left(V\right)=\delta \left(MP\left(A\nu gPool\left(V\right)\right)\right)+\delta \left(MP\left(MaxPool\left(V\right)\right)\right) \end{array}$$

where V is the input feature map, MP denotes the multilayer perceptron, and δ is the sigmoid activation function that ensures a non-linear transformation suitable for learning complex feature interdependencies.

SAM: This sub-module, on the other hand, captures the spatial relationships within the feature maps. By applying a convolutional operation across the spatial dimensions, it highlights critical areas of the image, ensuring that the model attends to significant regions, such as those indicating potential abnormalities in the lymph nodes:

(34) $$\begin{array}{c} N_{s}\left(V\right)=\delta \left(g^{7\times 7}\left[AvgPool\left(V\right);Max\left(Pool\left(V\right)\right)\right]\right) \end{array}$$

where $g^{7\times 7}$ is a convolution operation with a 7 × 7 kernel, focusing the model’s attention on spatial features that are clinically relevant. The CBAM module enhances the overall feature representation by sequentially applying CAM and SAM, resulting in more precise and informative feature maps. This attention-driven enhancement is crucial for accurately classifying lymph node ultrasound images, as it allows the model to focus on clinically relevant regions and ignore irrelevant background noise.

## 5. Implementation Details and Training Methodology

### 5.1. Dataset

To develop and validate our proposed deep learning model, we collected a comprehensive dataset of lymph node ultrasound images. A total of 1738 images were obtained from 1147 patients (age range: 20–60 years) at the Department of Ultrasound, Peking University Third Hospital, ensuring a balanced representation of both benign and malignant cases. Expert clinicians annotated the images, guaranteeing the high accuracy and reliability of the classification labels.

The dataset was preprocessed to enhance image quality and consistency, including cropping to remove irrelevant information, normalizing pixel values, and applying noise reduction techniques. The preprocessed dataset was then randomly split into training (70%, *n* = 1217), validation (15%, *n* = 260), and testing (15%, *n* = 261) sets, maintaining a similar distribution of benign and malignant cases across all sets.

### 5.2. Data Augmentation

One of the central challenges in developing deep learning models for medical imaging, particularly for rare or specialized conditions, is the scarcity of labeled data. This limitation can significantly hinder model training, as neural networks generally require large, diverse datasets to generalize well to new, unseen data. To mitigate this, we employed an extensive data augmentation strategy designed to artificially expand the available dataset while maintaining clinical relevance.

In addition to standard techniques such as affine transformations, rotation, and flipping, we introduced more advanced augmentation methods, including image mixing and noise simulation. Image mixing involves the combination of two ultrasound images, which allows the model to be exposed to novel feature combinations without losing critical diagnostic information. Gaussian noise was introduced to simulate ultrasound speckle, further enhancing the model’s ability to deal with real-world imaging variability. These augmentation techniques ensured that the model developed a robust understanding of both benign and malignant patterns in lymph nodes, allowing it to perform well across diverse imaging conditions. This involves carefully combining regions from different ultrasound images while preserving clinically relevant features. The image mixing technique can be described as

(35) $$\begin{array}{c} \tilde{x}=M⊙x_{A}+\left(1-M\right)⊙x_{B} \end{array}$$

where $\tilde{x}$ is the augmented image, $x_{A}$ and $x_{B}$ are two different ultrasound images, M is a binary mask, and ⊙ denotes element-wise multiplication. To simulate realistic imaging variations, we introduced noise and artifact simulation by adding Gaussian noise to mimic the ultrasound speckle pattern:

(36) $$\begin{array}{c} x_{\mathrm{noisy}}=x+\alpha \cdot N\left(0,\sigma^{2}\right) \end{array}$$

where $x_{\mathrm{noisy}\;}$ is the noisy image, x is the original image, α controls the noise intensity and $N\left(0,\sigma^{2}\right)$ represents Gaussian noise.

Inspired by the regional dropout strategy, we implemented local modifications, including selective masking and block replacement, to encourage the model to focus on multiple discriminative regions rather than relying on a single prominent feature. The masks for these regional modifications are generated as follows:

(37) $$\begin{array}{c} r_{x}∼Unif\left(0,W\right),r_{w}=W\sqrt{1-\lambda } \end{array}$$

(38) $$\begin{array}{c} r_{y}∼Unif\left(0,H\right),r_{h}=H\sqrt{1-\lambda } \end{array}$$

where $\left(r_{x},r_{y}\right)$ define the top-left corner of the modification region, and $\left(r_{w},r_{h}\right)$ define its width and height.

Additionally, we employed position jittering, introducing small random shifts in image position to enhance the model’s spatial invariance. Hybrid deep learning models for improving medical image classification accuracy have been presented. These augmentation methods are dynamically applied during training, creating a virtually expanded dataset that improves the model’s generalization ability and robustness to various imaging conditions.

All augmentations are meticulously calibrated to ensure that the images generated maintain clinical relevance and authenticity. For label mixing, we used the following formula:

(39) $$\begin{array}{c} \tilde{y}=\lambda y_{A}+\left(1-\lambda \right)y_{B} \end{array}$$

where $\tilde{y}$ is the new label, $y_{A}$ and $y_{B}$ are the original labels, and λ is the mixing ratio.

**Figure 5:**
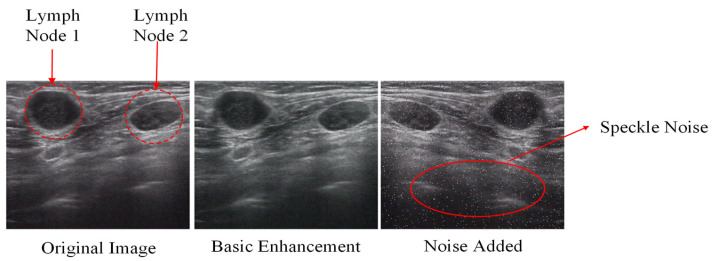
Ultrasound Image Augmentation: Original and Processed Comparisons.

Figure 5 provides a visual comparison between the original ultrasound images and their augmented counterparts, demonstrating how these techniques introduce variability while preserving the essential diagnostic features.

### 5.3. Training Strategy

The proposed model is primarily trained and validated on solid lymph nodes that display typical morphological features, such as shape, echogenicity, and margins, which are suggestive of benign or malignant processes. It is important to note that the model was not explicitly trained on other types of lymph nodes, such as cystic, calcified, or conglomerated lymph nodes. The scope of the current dataset is therefore limited to solid lymph nodes, and future work will expand to include additional lymph node types for broader applicability in clinical settings.

For model training, we adopted the AdamW optimizer. The application of attention mechanisms in lymph node image analysis to enhance diagnostic precision has been investigated, known for its adaptive learning rate capabilities. The initial learning rate was set to 5 × 10^−4^ and adjusted downward as training progressed. The update rule for the AdamW optimizer can be expressed as

(40) $$\begin{array}{c} \theta_{t+1}=\theta_{t}-\eta \cdot \frac{\hat{m_{t}}}{\sqrt{\hat{v_{t}}}+\epsilon }-\eta \cdot \lambda \cdot \theta_{t} \end{array}$$

where $\theta_{t}$ is the parameter at time step t, η is the learning rate, $\hat{m_{t}}$ and $\hat{v_{t}}$ are the bias-corrected first and second moment estimates, ϵ is a small constant for numerical stability, and λ is the weight decay parameter.

The loss function used was a combination of cross-entropy loss and an *L*_2_ regularization. Few-shot learning approaches and their potential in the field of medical imaging have been explored [25] to prevent overfitting. The total loss can be formulated as

(41) $$\begin{array}{c} L_{\mathrm{total}}=L_{\mathrm{CE}}+\lambda L_{2} \end{array}$$

where $L_{\mathrm{CE}}$ is the cross-entropy loss and $L_{2}$ is the *L*_2_ regularization term. The cross-entropy loss for binary classification is defined as

(42) $$\begin{array}{c} L_{\mathrm{CE}}=-\frac{1}{N}\overset{N}{\underset{i=1}{\sum }}\left[y_{i}\log\left({\hat{y}}_{i}\right)+\left(1-y_{i}\right)\log\left(1-{\hat{y}}_{i}\right)\right] \end{array}$$

where N is the number of samples, $y_{i}$ is the true label, and ${\hat{y}}_{i}$ is the predicted probability. The *L*_2_ regularization term is calculated as

(43) $$\begin{array}{c} L_{2}=\frac{1}{2}\sum_{w}w^{2} \end{array}$$

where w represents the model parameters.

The experiments were conducted in a controlled computational environment. The hardware setup included an NVIDIA GTX 1080 Ti GPU (NVIDIA Corporation, Santa Clara, CA, USA) with 8 GB of memory, which facilitated efficient training of the deep learning model. The software environment comprised the Windows 10 operating system, PyCharm IDE (2024.4), and the PyTorch deep learning framework (version 1.11.0), with Python 3.6 as the programming language.

We trained the model in batches of 32 images over the course of 500 epochs, balancing the need for sufficient iterations to allow the model to learn complex patterns while preventing overfitting. The batch size of 32 was selected to achieve a trade-off between computational efficiency and convergence stability, ensuring that each batch provided diverse data for the model to learn from while keeping memory usage within the limits of our hardware. Each epoch involved a full pass through the training dataset, progressively refining the model’s parameters through backpropagation. To ensure optimal learning, we monitored the model’s performance on a validation set after each epoch, which provided an unbiased assessment of its generalization capabilities. Key performance metrics, such as validation accuracy, loss, and accuracy, were closely observed, allowing us to make dynamic adjustments to the learning rate and apply regularization techniques to mitigate the risk of overfitting. Overfitting, a common issue in deep learning, where the model memorizes the training data rather than learning general patterns, was countered by using techniques such as early stopping and learning rate decay. These strategies ensured that the model did not train for too long or with a learning rate that could lead to poor generalization. At the end of the training process, we employed model checkpointing, retaining the model parameters that demonstrated the best performance on the validation set. This approach ensured that the final model was not only highly accurate on the training data but also able to generalize effectively to new, unseen data, making it well-suited for real-world applications, where variability in ultrasound images is expected.

The NVIDIA GTX 1080 Ti GPU provided the necessary computational power to handle the large dataset and complex model architecture. PyTorch was chosen for its flexibility and ease of use in implementing deep learning models. The AdamW optimizer was selected due to its ability to adaptively adjust learning rates, which helps in achieving faster convergence and better performance. The use of cross-entropy loss with *L*_2_ regularization ensured that the model could effectively handle the classification task while avoiding overfitting, which is a common issue in deep learning. The training process required approximately 3 min per image with a batch size of 32 and a resolution of 256 × 256 on the NVIDIA GTX 1080 Ti GPU. This training time represents a balance between computational efficiency and model performance, ensuring practical feasibility for clinical implementation.

### 5.4. Validation Strategy

**Figure 6:**
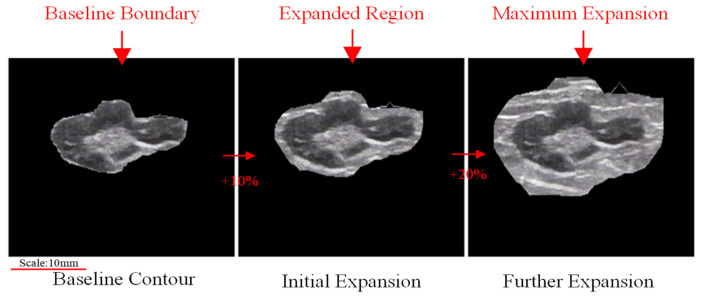
Expanded outline diagram.

We employed a comparative experimental approach using 10-fold cross-validation to evaluate model performance across various dataset configurations. These configurations included the original dataset, an augmented dataset (using techniques like rotation, scaling, and flipping), and an externally expanded lymph node [26] dataset. The expanded dataset was tested with three expansion ratios: the baseline boundary (10%), one-level expansion (20%), and maximum expansion (30%), as illustrated in Figure 6, where red arrows indicate the progression between each expansion stage. For each configuration, the dataset was randomly divided into 10 equal subsets, with 9 used for training and 1 for validation in each fold. This process was repeated 10 times, with performance metrics averaged across all folds. This comprehensive approach allowed us to compare model performances under different conditions and data modifications, ultimately aiding in identifying the optimal model and dataset combination for lymph node classification.

## 6. Experimental Results and Analysis

There are three targets taken to evaluate the performance of the lymph node classification model used in this article. They are True Negative Rate (TNR) for specificity, True Positive Rate (TPR) for sensitivity, and Accuracy (ACC) indicators, which are defined as follows:

(44) $$\begin{array}{c} TNR=\frac{TN}{TN+FP},TPR=\frac{TP}{TP+FN} \end{array}$$

(45) $$\begin{array}{c} FPR=\frac{FP}{FP+TN},ACC=\frac{TP+TN}{TP+TN+FP+FN} \end{array}$$

where TP, TN, FP, and FN are the numbers of true positives, true negatives, false positives, and false negatives, respectively. Generally speaking, high specificity means low misdiagnosis rate, and high sensitivity means low missed diagnosis rate. The higher the accuracy, the better the classification effect.

### 6.1. Classification Results on Different Datasets

To evaluate the performance of our proposed DSC-Transformer model, we conducted extensive experiments on three types of datasets: original, augmented, and externally expanded datasets [27,28,29].

The currently used common classification models are ResNet34, MobileNet, DenseNet, Vision Transformer, Swin Transformer, ConvNext, etc. [30,31]. Table 1 presents the experimental results for each classification model obtained using the original dataset. The experimental results for each classification model of the dataset after data enhancement are depicted in Table 2.

**Figure 7:**
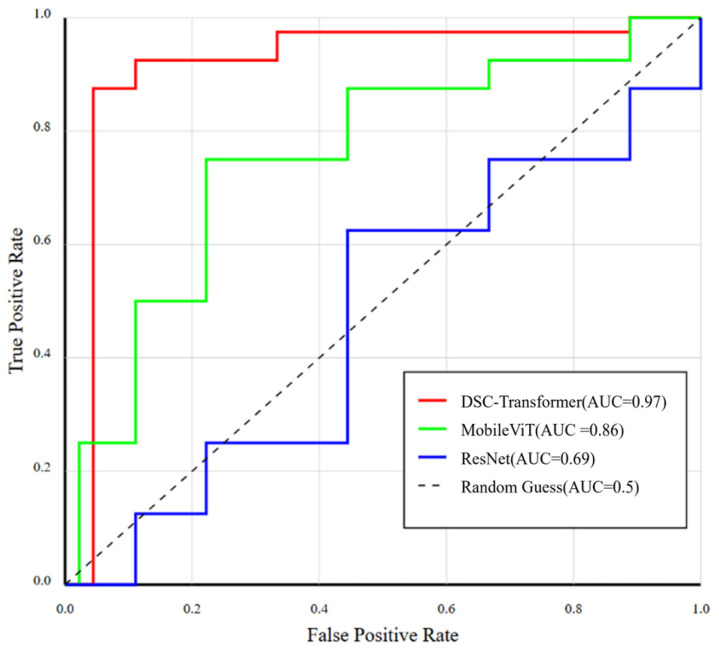
ROC analysis curves of different models.

The Receiver Operating Characteristics ROC curves of the different models in the task of classifying ultrasound images of lymph nodes are illustrated in Figure 7. The curve of SC-Transformer is closest to the upper left corner, with an AUC value of 0.97, which is significantly better than the other models. MobileViT performs next best with an AUC of 0.86, while the ResNet has an AUC of 0.69. These results clearly demonstrate the superiority of DSC-Transformer in terms of classification performance, in particular the significant reduction in the false positive rate while maintaining a high true positive rate. This performance enhancement can be attributed to the fact that SC-Transformer combines the hierarchical structure of Swin Transformer with the attention mechanism of CBAM, which enhances the ability to extract and focus on clinically relevant features [37]. To further validate the performance of the model on different datasets, we conducted a series of data enhancement and extension experiments. A single lymph node dataset is expanded typically by using two methods. One approach is to expand based on the minimum bounding rectangle, whereas the other approach is to expand based on the image contour. The dataset can be expanded based on the minimum bounding rectangle by using the OpenCV library. By obtaining the coordinates of the bounding rectangle, the individual lymph node can be segmented and extracted. This method provides a precise and rectangular region for each lymph node.

**Figure 8:**
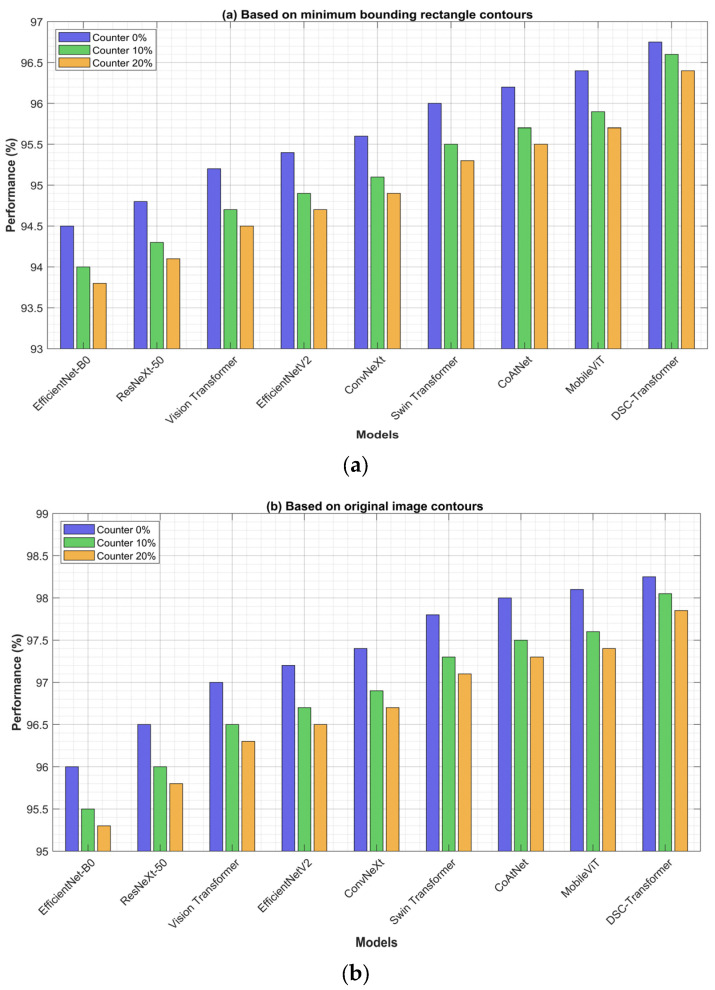
Experimental results of scaling up for different datasets. (**a**) Based on the lymph nodes’ minimum bounding rectangle contours. (**b**) Based on the original image contours.

When expanding the dataset based on the image contour, the process involves treating the image as a polygon, which captures the precise outline of the lymph node or other anatomical structures. This method, though effective in preserving the exact shape of the object, can occasionally result in slightly less smooth boundaries with some curvature after the expansion. However, these minor shape irregularities have a negligible impact on the overall experiment and can largely be disregarded in terms of their influence on model performance [38]. In Figure 8a, we present the experimental results of dataset expansion using the lymph nodes’ minimum bounding rectangle contours, which serve to simplify the contour representation by enclosing the lymph node within the smallest possible rectangle [39]. This approach ensures that no critical image information is lost, while providing a standardized way to expand the dataset.

The classification results across different datasets—both augmented and non-augmented—highlight the clear effectiveness of our data augmentation strategies. The most significant improvements in model performance were observed on the augmented dataset, demonstrating the critical role that data augmentation plays in enhancing the model’s learning capabilities [40]. By exposing the model to a broader and more diverse set of training examples, we enable it to learn more robust and generalizable features. This process helps the model better capture the variations in lymph node images, such as subtle differences in shape, texture, and size, which are essential for distinguishing between benign and malignant cases.

The improvements in classification accuracy on the augmented dataset clearly underscore the importance of introducing variability during the training phase. These augmented images serve to simulate real-world variations, effectively preventing overfitting and allowing the model to generalize more effectively to unseen data. Figure 8b illustrates the experimental results of dataset expansion based on the original image contours, which provide a more detailed representation of the lymph node’s shape compared to the rectangular bounding approach. Despite the increased complexity in contour representation, the experimental results consistently show that both expansion methods contribute significantly to enhancing the model’s overall classification performance, reinforcing the value of diverse data augmentation techniques in medical image analysis.

**Figure 9:**
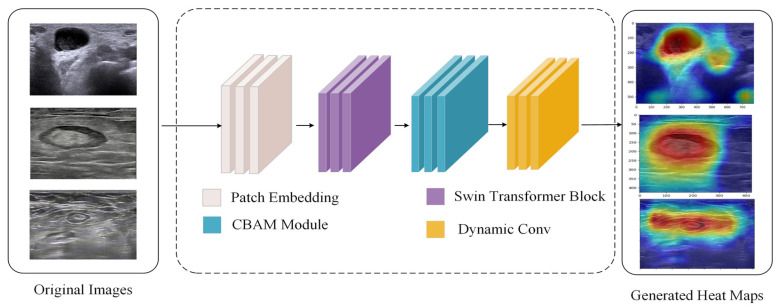
Depiction of the heat map.

The dataset with data enhancement exhibited the best effect. The Grad-CAM visualization tool (version 1.4.6) was used to visualize the feature map and generate heatmaps to further explore the effectiveness of the classification network. In terms of real-time performance, the model achieves a processing speed of 25 frames per second (FPS) on the NVIDIA GTX 1080 Ti GPU, meeting the requirements for real-time clinical applications. This frame rate ensures smooth and efficient processing of ultrasound images during live examinations, enabling immediate feedback for healthcare providers while maintaining the model’s high classification accuracy. As shown in Figure 9, our model processes the ultrasound images through four key stages: patch embedding, Swin Transformer block, CBAM module, and dynamic convolution. The generated heat maps (right panel) demonstrate the model’s attention regions, where warmer colors (red) indicate areas of higher attention while cooler colors (blue) represent areas of lower attention. The model accurately focuses on the internal structures of lymph nodes, as evidenced by the concentrated red regions in the heat maps. This attention pattern aligns well with clinical diagnostic requirements, confirming that our model has developed precise feature identification capabilities for lymph node classification.

### 6.2. Ablation Studies

To elucidate the individual and combined contributions of key components within the DSC-Transformer model, we conducted comprehensive ablation studies [35,36]. These studies systematically evaluated four configurations: the full model, the model without dynamic convolution, the model without CBAM, and the model with both components removed. The results of these experiments are summarized in Table 3.

The full DSC-Transformer model, incorporating both dynamic convolution and CBAM, achieved the highest performance across all metrics. Removing the dynamic convolution module resulted in a moderate performance decrease, with accuracy reducing to 97.37%. Exclusion of the CBAM module led to performance degradation, with accuracy decreasing to 96.81%. Removing both components resulted in the most significant performance decline, with accuracy falling to 95.60%.

**Figure 10:**
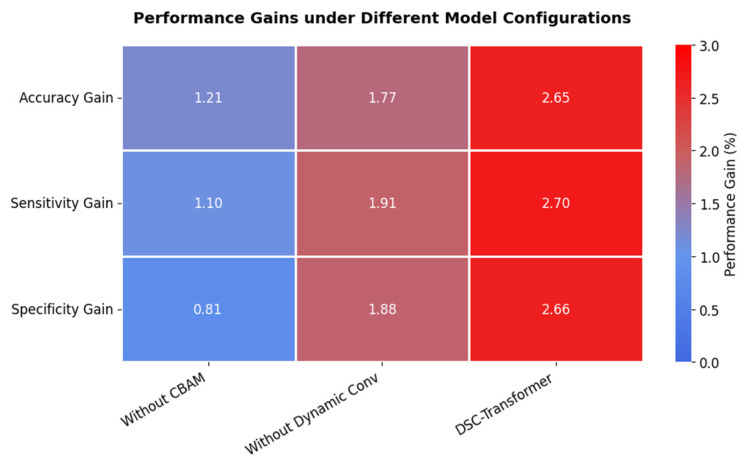
Performance Gains under Different Model Configurations Relative to Base Model.

The ablation study revealed progressive improvements with each component addition. The full DSC-Transformer achieved gains of 2.65%, 2.70%, and 2.66% in accuracy, sensitivity, and specificity respectively compared to the base model, demonstrating the synergistic effect of combining both components. Figure 10 shows these performance gains through a heat map visualization, where darker colors indicate higher gains achieved by the full model.

Beyond raw accuracy, the DSC-Transformer excelled in sensitivity (98.05%) and specificity (98.17%), which are crucial metrics for lymph node classification where both false negatives and false positives can have serious clinical implications. The high performance across all metrics demonstrates its potential as a robust tool for automated lymph node classification in clinical settings.

The methodology has significant promise in the timely identification and categorization of lymph node irregularities. The high precision, sensitivity, and specificity of this technology can aid physicians in identifying illnesses such as cancer and infections with greater efficiency and accuracy. The model’s capacity to differentiate between benign and malignant lymph nodes might minimize the likelihood of misdiagnosis, guaranteeing prompt and suitable treatment. This model has the capability to automate the diagnostic process, which can reduce the burden of healthcare personnel, simplify clinical procedures, and ultimately enhance patient outcomes.

### 6.3. Impact of Semantic Communication

**Figure 11:**
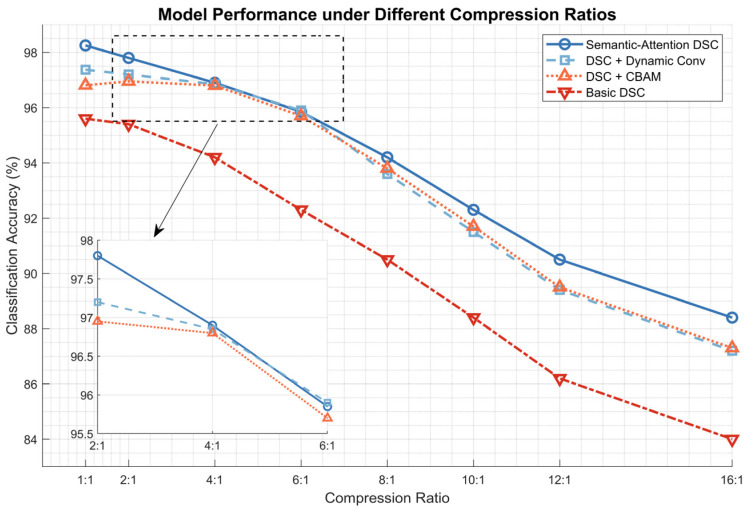
Performance Comparison of Semantic-Attention Enhanced DSC-Transformer under Different Compression Ratios.

The effectiveness of semantic communication in our proposed model was evaluated through extensive experiments focusing on the relationship between compression ratios and classification performance. Figure 11 illustrates the model’s performance under different compression scenarios, revealing several key insights into the impact of semantic communication.

The experimental results demonstrate that, as the compression ratio increases from 1:1 to 16:1, all models show a decline in classification accuracy, but with notably different degradation patterns. The Semantic-Attention DSC model maintains superior performance across all compression ratios, achieving 98.25% accuracy at 1:1 compression and maintaining high performance (96.90%) even at 4:1 compression. At moderate compression ratios (2:1 to 6:1), as shown in the zoomed view, while DSC + Dynamic Conv and DSC + CBAM show similar performance patterns with intersecting accuracies, the Semantic-Attention DSC consistently maintains higher accuracy, demonstrating the effectiveness of integrated semantic communication. This advantage becomes more pronounced at higher compression ratios (8:1 to 16:1), where the model maintains accuracy above 88.40% while the basic DSC model drops to 84.00%. This robustness can be attributed to the model’s semantic feature extraction and attention mechanisms, which effectively preserve diagnostically relevant information while reducing data volume.

The superior performance of the Semantic-Attention Enhanced DSC-Transformer, especially under high compression scenarios, demonstrates the effectiveness of combining semantic communication with attention mechanisms for remote medical image analysis. This approach not only reduces transmission bandwidth requirements but also ensures the preservation of critical diagnostic information, making it a promising solution for practical telemedicine applications.

## 7. Discussion and Future Directions

### 7.1. Strengths of the Proposed Approach

The Semantic-Attention Enhanced DSC-Transformer demonstrates several significant advantages in addressing the challenges of remote lymph node ultrasound diagnostics. Firstly, the integration of semantic communication significantly reduces bandwidth requirements while maintaining diagnostic accuracy, achieving 96.90% accuracy even at 4:1 compression ratios. This capability is particularly valuable in resource-constrained healthcare settings where bandwidth limitations often impede remote diagnostics.

The model’s dual-attention mechanism combined with dynamic convolution provides superior feature extraction capabilities, enabling precise identification of diagnostically relevant regions within ultrasound images. This is evidenced by the model’s high classification accuracy (98.25%), sensitivity (98.05%), and specificity (98.17%) on the augmented dataset, surpassing traditional approaches. The effectiveness of this approach is particularly apparent in the Grad-CAM visualizations, which demonstrate the model’s ability to focus on clinically significant regions of lymph nodes.

Although the focus of our current experiments is on classifying a specific group of lymph nodes, in particular tumor types, such as those associated with breast and pelvic cancers, we acknowledge that the proposed method can be extended to broader malignant lymph node evaluations. Additionally, while our model currently classifies lymph nodes into benign and malignant categories, future work could explore multi-class classification, such as distinguishing benign, malignant, and inflammatory conditions, assuming sufficient labeled datasets are available. This expansion would allow our approach to handle a broader spectrum of lymph node pathologies, including non-malignant granulomatous or inflammatory conditions like tuberculosis or sarcoidosis, which sometimes share imaging features with malignant lymph nodes. The methodology is not inherently limited to just a few tumor categories and can be adapted for various clinical settings where lymph node assessment is critical [40].

Furthermore, the semantic preprocessing pipeline enhances the model’s adaptability to varying image qualities and conditions, making it particularly suitable for real-world clinical applications. The hierarchical feature extraction process, combined with semantic compression, ensures that critical diagnostic information is preserved even under challenging transmission conditions, addressing a key concern in telemedicine applications.

### 7.2. Limitations and Challenges

Despite its impressive performance, the proposed approach faces several notable limitations and challenges. The primary challenge lies in the complexity of semantic feature extraction, which requires significant computational resources during the initial processing phase. While this is offset by reduced transmission requirements, it may pose implementation challenges in resource-limited clinical settings.

The current model’s performance, though strong, relies heavily on high-quality training data. The availability of diverse, well-annotated lymph node ultrasound images remains limited, potentially affecting the model’s generalization capabilities across different patient populations and imaging conditions. Additionally, the model’s performance under extreme compression ratios (beyond 8:1) shows some degradation, indicating room for improvement in semantic preservation at higher compression levels.

Another significant challenge is the need for real-time processing capabilities in clinical settings. While the model achieves excellent accuracy, optimizing its inference speed for real-time applications while maintaining high precision remains an important area for improvement.

One additional limitation of the current model is its handling of conglomerated or packeted lymph nodes. In cases where multiple lymph nodes are grouped together, distinguishing individual boundaries can be challenging, potentially leading to reduced accuracy in classification. This issue has not been addressed explicitly in the model’s current training, which primarily focuses on solid lymph nodes. Expanding the model to handle such cases, along with other types of lymph nodes like cystic or calcified forms, will require the acquisition of annotated datasets specific to these categories. Future research will focus on collecting these datasets and extending the model’s capabilities to cover a broader range of lymph node types, ensuring better accuracy across diverse clinical scenarios.

While the model’s computational complexity presents challenges for local implementation, these limitations can be effectively addressed through cloud-based deployment. A hybrid edge-cloud architecture enables the semantic preprocessing to run on edge devices while leveraging cloud resources for computationally intensive operations. This approach not only resolves hardware constraints but also provides scalability and accessibility benefits for healthcare providers. Cloud deployment allows for dynamic resource allocation based on demand, making the system more cost-effective and practical for clinical implementation.

## 8. Conclusions

This study introduces a novel Semantic-Attention Enhanced DSC-Transformer for lymph node ultrasound image classification, integrating the Swin Transformer architecture with dynamic convolution and CBAM. Through comprehensive experimentation and ablation studies, our model demonstrates superior performance in classification accuracy, sensitivity, and specificity compared to existing approaches. The integration of semantic communication with attention mechanisms enables efficient data transmission while preserving diagnostic accuracy, making it particularly valuable for remote healthcare applications. The model’s ability to focus on clinically relevant regions, validated through Grad-CAM visualizations, enhances its practical utility in clinical settings. This advancement in medical image analysis contributes significantly to improving early detection and diagnosis of lymph node abnormalities, particularly in resource-constrained environments where efficient remote diagnostics are crucial.

## Figures and Tables

**Table 1:** Each Classification Model Using the Original Dataset.

Network	Size (MB)	Speed (ms/im)	Acc (%)	Sensitivity (%)	Specificity (%)	F1	AUC
EfficientNet-B0 [23]	20	15	94.5	94.0	94.8	0.94	0.97
ResNeXt-50 [24]	98	25	94.8	94.3	95.1	0.95	0.98
Vision Transformer [26]	86	30	95.2	94.7	95.5	0.96	0.98
EfficientNetV2 [27]	24	18	95.4	94.9	95.7	0.95	0.98
ConvNeXt [32]	89	22	95.6	95.1	95.9	0.96	0.99
Swin Transformer [33]	88	28	96.0	95.5	96.3	0.96	0.99
CoAtNet [34]	96	26	96.2	95.7	96.5	0.96	0.98
MobileViT [35]	5.7	12	96.4	95.9	96.7	0.96	0.97
Masked Autoencoder [36]	86	32	96.6	96.1	96.9	0.97	0.99
DSC-Transformer	43.6	20	96.75	96.60	98.06	0.97	0.99

**Table 2:** Experimental Results After Enhancement of Each Classification Model for the Dataset.

Network	Acc (%)	Sensitivity (%)	Specificity (%)	F1	AUC
EfficientNet-B0 [23]	96.0	95.5	96.3	0.95	0.93
ResNeXt-50 [24]	96.5	96.0	96.8	0.96	0.94
Vision Transformer [26]	97.0	96.5	97.3	0.93	0.95
EfficientNetV2 [27]	97.2	96.7	97.5	0.95	0.96
ConvNeXt [32]	97.4	96.9	97.7	0.97	0.98
Swin Transformer [33]	97.8	97.3	98.1	0.97	0.99
CoAtNet [34]	98.0	97.5	98.3	0.96	0.98
MobileViT [35]	98.1	97.6	98.4	0.96	0.96
Masked Autoencoder [36]	98.2	97.7	96.5	0.97	0.98
DSC-Transformer	98.25	98.05	98.17	0.98	0.99

**Table 3:** Results of The Ablation Study.

Model Configuration	Accuracy (%)	Sensitivity (%)	Specificity (%)
Without Both	95.60	95.35	95.51
Without CBAM	96.81	96.45	96.32
Without Dynamic Conv	97.37	97.26	97.39
DSC-Transformer	98.25	98.05	98.17

## Data Availability

The original contributions presented in the study are included in the article; further inquiries can be directed to the corresponding author.

## References

[1] Zhou B., Yang X., Curran W.J., Tian L. (2022). Artificial intelligence in quantitative ultrasound imaging: A survey. J. Ultrasound Med..

[2] Rochlin D.H., Barrio A.V., McLaughlin S. (2023). Feasibility and clinical utility of prediction models for breast cancer–related lymphedema incorporating racial differences in disease incidence. JAMA Surg..

[3] Wild S.R., Koppert L.B., Nijnatten T.J.A. (2024). Systematic review of targeted axillary dissection in node-positive breast cancer treated with neoadjuvant systemic therapy: Variation in type of marker and timing of placement. Br. J. Surg..

[4] Liu S., Fu Y., Cui L., Wang S., Tan S. (2024). Role of Ultrasonography in Monitoring Chemotherapeutic Effects on Primary Thyroid Lymphoma: A Single-Center Retrospective Study. Medicina.

[5] Alabousi M., Alabousi A., Adham S., Pozdnyakov A., Ramadan S., Chaudhari H., Harish S. (2022). Diagnostic test accuracy of ultrasonography vs computed tomography for papillary thyroid cancer cervical lymph node metastasis: A systematic review and meta-analysis. JAMA Otolaryngol. Head. Neck. Surg..

[6] Zhuang Z., Zhang Y., Wei M., Yang X., Wang Z. (2021). Magnetic resonance imaging evaluation of the accuracy of various lymph node staging criteria in rectal cancer: A systematic review and meta-analysis. Front. Oncol..

[7] Garganese G., Bove S., Fragomeni S., Moro F., Triumbari E.K.A., Collarino A., Verri D., Gentileschi S., Sperduti I., Scambia G. (2021). Real-time ultrasound virtual navigation in 3D PET/CT volumes for superficial lymph-node evaluation: Innovative fusion examination. Ultrasound Obstet. Gynecol..

[8] Dilege E., Celik B., Falay O., Boge M., Sucu S., Toprak S., Agcaoglu O., Kapucuoglu N., Demirkol O. (2023). SPECT/CT lymphoscintigraphy accurately localizes clipped and sentinel nodes after neoadjuvant chemotherapy in node-positive breast cancer. Clin. Nucl. Med..

[9] Olthof E.P., van der Aa M.A., Adam J.A., Stalpers L.J., Wenzel H.H., van der Velden J., Mom C.H. (2021). The role of lymph nodes in cervical cancer: Incidence and identification of lymph node metastases—A literature review. Int. J. Clin. Oncol..

[10] Khan A., Khan S.H., Saif M., Batool A., Sohail A., Waleed Khan M. (2023). A Survey of Deep Learning Techniques for the Analysis of COVID-19 and their usability for Detecting Omicron. J. Exp. Theor. Artif. Intell..

[11] Pandey A., Pandey Y. Multi-Level Feature-Based CNN Model for Tumor Segmentation Algorithm. Proceedings of the 2023 IEEE 20th India Council International Conference (INDICON).

[12] Berroukham A., Housni K., Lahraichi M. Vision Transformers: A Review of Architecture, Applications, and Future Directions. Proceedings of the 2023 7th IEEE Congress on Information Science and Technology (CiSt).

[13] Abdellatif A., Mubarak H., Ahmad S., Mekhilef S., Abdellatef H., Mokhlis H., Kanesan J. Electricity Price Forecasting One Day Ahead by Employing Hybrid Deep Learning Model. Proceedings of the 2023 IEEE IAS Global Conference on Renewable Energy and Hydrogen Technologies (GlobConHT).

[14] Li J., Liu H., Li K., Shan K. Heart Sound Classification Based on Two-channel Feature Fusion and Dual Attention Mechanism. Proceedings of the 2024 5th International Conference on Computer Engineering and Application (ICCEA).

[15] Jin J., Wang F. Meta Learning-Based Approach for Few-Shot Target Recognition in ISAR Images. Proceedings of the IGARSS 2023—2023 IEEE International Geoscience and Remote Sensing Symposium.

[16] Shivwanshi R.R., Nirala N. Implementation of an advanced lung nodule classification system using optimized ConvMixer and AdamW-based CNN architecture. Proceedings of the 2023 Signal Processing: Algorithms, Architectures, Arrangements, and Applications (SPA).

[17] Li S., Tu Y., Xiang Q., Li Z. MAGIC: Rethinking Dynamic Convolution Design for Medical Image Segmentation. Proceedings of the 32nd ACM International Conference on Multimedia.

[18] Shin Y., Kim S. Infrared Pedestrian Dataset Training using Swin Transformer model. Proceedings of the 2022 22nd International Conference on Control, Automation and Systems (ICCAS).

[19] Mohanty S.P., Hughes D.P., Salathé M. (2016). Using deep learning for image-based plant disease detection. Front. Plant Sci..

[20] Dosovitskiy A., Beyer L., Kolesnikov A., Weissenborn D., Zhai X., Unterthiner T., Dehghani M., Minderer M., Heigold G., Gelly S. (2020). An image is worth 16×16 words: Transformers for image recognition at scale. arXiv.

[21] Kumar E.S., Bindu C.S. Segmentation of Retinal Lesions in Fundus Images: A Patch Based Approach Using Encoder-Decoder Neural Network. Proceedings of the 2021 7th International Conference on Advanced Computing and Communication Systems (ICACCS).

[22] Xie S., Girshick R., Dollár P., Tu Z., He K. Aggregated residual transformations for deep neural networks. Proceedings of the IEEE Conference on Computer Vision and Pattern Recognition.

[23] Yun S., Han D., Oh S.J., Chun S., Choe J., Yoo Y. CutMix: Regularization Strategy to Train Strong Classifiers with Localizable Features. Proceedings of the 2019 IEEE/CVF International Conference on Computer Vision (ICCV).

[24] Wu M., Yan C., Wang X., Liu Q., Liu Z., Song T. (2022). Automatic classification of hepatic cystic echinococcosis using ultrasound images and deep learning. J. Ultrasound Med..

[25] Haddadi Y.R., Mansouri B. Ultrasound Medical Image Deconvolution Using *L*_2_ Regularization Method and Artificial Bee Colony Optimization Algorithm. Proceedings of the 2022 7th International Conference on Image and Signal Processing and their Applications (ISPA).

[26] Glaser S., Maicas G., Bedrikovetski S., Sammour T., Carneiro G. Semi-Supervised Multi-Domain Multi-Task Training for Metastatic Colon Lymph Node Diagnosis from Abdominal CT. Proceedings of the 2020 IEEE 17th International Symposium on Biomedical Imaging (ISBI).

[27] Fu Y., Shi Y.F., Yan K., Wang Y.J., Yang W., Feng G.S. (2014). Clinical value of real time elastography in patients with unexplained cervical lymphadenopathy: Quantitative evaluation. Asian Pac. J. Cancer Prev..

[28] Liu Z., Mao H., Wu C.Y., Feichtenhofer C., Darrell T., Xie S. A ConvNet for the 2020s. Proceedings of the IEEE/CVF Conference on Computer Vision and Pattern Recognition.

[29] Fang F., Hu X., Shu J., Wang P., Shen T., Li F. Text Classification Model Based on Multi-head self-attention mechanism and BiGRU. Proceedings of the 2021 IEEE Conference on Telecommunications, Optics and Computer Science (TOCS).

[30] Iadecola C. (2017). The Neurovascular Unit Coming of Age: A Journey through Neurovascular Coupling in Health and Disease. Neuron.

[31] Oduro-Gyimah F.K., Boateng K.O., Adu P.B., Quist-Aphetsi K. Prediction of Telecommunication Network Outage Time Using Multilayer Perceptron Modelling Approach. Proceedings of the 2021 International Conference on Computing, Computational Modelling and Applications (ICCMA).

[32] Cheng A., Xiao J., Li Y., Sun Y., Ren Y., Liu J. (2024). Enhancing Remote Sensing Object Detection with K-CBST YOLO: Integrating CBAM and Swin-Transformer. Remote Sens..

[33] Wang H., Yu G., Cheng J., Zhang Z., Wang X., Xu Y. (2024). Fast Hyperspectral Image Classification with Strong Noise Robustness Based on Minimum Noise Fraction. Remote Sens..

[34] Song W., Nie F., Wang C., Jiang Y., Wu Y. (2024). Unsupervised Multi-Scale Hybrid Feature Extraction Network for Semantic Segmentation of High-Resolution Remote Sensing Images. Remote Sens..

[35] Fu Y., Cui L.G., Ma J.Y., Fang M., Lin Y.X., Li N. (2024). Development of a Novel Contrast-Enhanced Ultrasound-Based Nomogram for Superficial Lymphadenopathy Differentiation: Postvascular Phase Value. Ultrasound Med. Biol..

[36] Sathish S.H. (2023). Emotions Recognition Using Multimodal Spontaneous Emotion Database and Deep Learning Technology. Master’s Thesis.

[37] Mehta S., Rastegari M. (2021). MobileViT: Light-weight, general-purpose, and mobile-friendly vision transformer. arXiv.

[38] He K., Chen X., Xie S., Li Y., Dollár P., Girshick R. Masked autoencoders are scalable vision learners. Proceedings of the IEEE/CVF Conference on Computer Vision and Pattern Recognition.

[39] Zhang X., Zhao R., Wu X., Mu W. (2022). Hydrogeochemistry, identification of hydrogeochemical evolution mechanisms, and assessment of groundwater quality in the southwestern Ordos Basin, China. Environ. Sci. Pollut. Res..

[40] Al-Abbadi M.A., Barroca H., Bode-Lesniewska B., Calaminici M., Caraway N.P., Chhieng D.F., Cozzolino I., Ehinger M., Field A.S., Geddie W.R. (2020). A proposal for the performance, classification, and reporting of lymph node fine-needle aspiration cytopathology: The Sydney system. Acta Cytol..

